# Spectral-temporal EEG dynamics of speech discrimination processing in infants during sleep

**DOI:** 10.1186/s12868-017-0353-4

**Published:** 2017-03-22

**Authors:** Phillip M. Gilley, Kristin Uhler, Kaylee Watson, Christine Yoshinaga-Itano

**Affiliations:** 1 Institute of Cognitive Science, University of Colorado, Boulder, Boulder, CO USA; 2 Marion Downs Center, Denver, CO USA; 3 University of Colorado School of Medicine, Aurora, CO USA; 4 Department of Speech, Language, and Hearing Sciences, University of Colorado, Boulder, Boulder, CO USA

**Keywords:** EEG, Auditory evoked potentials, Mismatch negativity, Speech discrimination, Infants, Sleep, Surprise

## Abstract

**Background:**

Oddball paradigms are frequently used to study auditory discrimination by comparing event-related potential (ERP) responses from a standard, high probability sound and to a deviant, low probability sound. Previous research has established that such paradigms, such as the mismatch response or mismatch negativity, are useful for examining auditory processes in young children and infants across various sleep and attention states. The extent to which oddball ERP responses may reflect subtle discrimination effects, such as speech discrimination, is largely unknown, especially in infants that have not yet acquired speech and language.

**Results:**

Mismatch responses for three contrasts (non-speech, vowel, and consonant) were computed as a spectral-temporal probability function in 24 infants, and analyzed at the group level by a modified multidimensional scaling. Immediately following an onset gamma response (30–50 Hz), the emergence of a beta oscillation (12–30 Hz) was temporally coupled with a lower frequency theta oscillation (2–8 Hz). The spectral-temporal probability of this coupling effect relative to a subsequent theta modulation corresponds with discrimination difficulty for non-speech, vowel, and consonant contrast features.

**Discussion:**

The theta modulation effect suggests that unexpected sounds are encoded as a probabilistic measure of surprise. These results support the notion that auditory discrimination is driven by the development of brain networks for predictive processing, and can be measured in infants during sleep. The results presented here have implications for the interpretation of discrimination as a probabilistic process, and may provide a basis for the development of single-subject and single-trial classification in a clinically useful context.

**Conclusion:**

An infant’s brain is processing information about the environment and performing computations, even during sleep. These computations reflect subtle differences in acoustic feature processing that are necessary for language-learning. Results from this study suggest that brain responses to deviant sounds in an oddball paradigm follow a cascade of oscillatory modulations. This cascade begins with a gamma response that later emerges as a beta synchronization, which is temporally coupled with a theta modulation, and followed by a second, subsequent theta modulation. The difference in frequency and timing of the theta modulations appears to reflect a measure of surprise. These insights into the neurophysiological mechanisms of auditory discrimination provide a basis for exploring the clinically utility of the *MMR*_*TF*_ and other auditory oddball responses.

## Background

Speech discrimination inherently requires a comparison of two or more different sounds, which, in EEG studies, is typically assessed by some variant of an oddball response task [cf., 1, 2], and elicits an event-related potential (ERP) in the EEG signal. An oddball task includes the repeated presentation of two sounds^1^ over many trials with some associated probability of which sound is selected during a trial. The sound with the highest probability of selection (i.e., the sound heard most often) is referred to as the “standard” or “frequent” stimulus, while the sound with the lowest probability is called the “deviant” or “rare” stimulus. If the task is meant to assess pre-perceptual processing, then the participant is usually asked to ignore the sounds and/or to attend to a separate task. Because these oddball responses can be assessed in the absence of a behavioral component, such a task might provide a solution to assessing discrimination in young infants.

Pre-perceptual oddball tasks are presumed to elicit an automatic, exogenous change-detection response [3]. For example, the mismatch negativity or mismatch response^2^ (MMR/N) has been well defined in adults [4–6] and children [7–10], and has been correlated with behavioral discrimination [11, 12]. As the MMR/N is elicited passively and in the absence of directed attention [1, 13, 14], it has considerable potential as a measure of early information processing in infants [15]. Although considered a pre-attentive response, there has been debate on the modulatory effects of attention on the response [16]. Studies with adults attempt to control attention by having participants attend a visual stimulus during testing, but controlling attention in awake/alert infants may be more problematic. Conversely, studies in infants report stability in key ERP parameters (latency, amplitude, etc.) across various stages of alertness, including sleep [9, 17, 18]. Testing during sleep not only reduces the potential influence of attention effects, it has the added benefit of reducing data loss associated with movement artifacts.

During sleep, EEG in young children contains more low frequency power in the delta (2–4 Hz) and theta (4–8 Hz) frequency bands compared to older children and adults [19]. However, the exact contributions of these frequency bands also vary by sleep stage [20], and some infants (~35%) may exhibit transient theta-alpha (8–12 Hz) bursts in the EEG [21]. Despite such variance in sleep-stage contributions, auditory ERPs reveal typical auditory processing patterns during sleep and through the sleep-wake cycle [20]. However, detecting ERP differences in higher frequency bands, such as beta (12–30 Hz) and gamma (30–50 Hz) can be challenging given the smaller contributions to the EEG and unknown variability at those higher frequencies.

Several studies have now demonstrated theta amplitude modulations (AM) for frontal MMR/N components concurrent with theta phase modulations (PM) for temporal MMR/N components (i.e., as observed over temporal cortex) when detecting a deviant stimulus [22–24]. These theta modulations also appear to follow a series of modulations in the gamma band (including high gamma, 60–300 Hz) and may be related to inhibitory changes in the alpha band [25]. Taken together, these previous results suggest a dynamical relationship between different neurophysiological mechanisms that contribute to the detection of a deviant sound. In the present study, we examine two hypotheses: (1) spectral-temporal features of auditory discrimination can be extracted from a “single-channel” montage that is ideal for clinical application in infants, and (2) these spectral-temporal features reflect the degree of difficulty for discriminating two sounds.

## Methods

### Participants

Participants for this study included 24 typically developing infants (10 female, 14 male) aged 1.0–3.9 months (mean = 2.6, SD = 0.82). All infants had passed their newborn hearing screening assessed by a click evoked auditory brainstem response (ABR) screening protocol. The informed consent form was fully executed prior to any study related activity, as approved by the Colorado Multiple Institutional Review Board.

### Stimuli

Three stimulus pairs, or “contrasts” were presented in separate blocks using a standard auditory oddball paradigm with an inter-stimulus interval (ISI) of 1200 ms. This long interval increases the likelihood of identifying an auditory evoked response in young children [26, 27]. Stimuli were presented in pseudo-random order at a ratio of 85% *standard* to 15% *deviant*, with the constraint that deviant stimuli could not appear in succession. Approximately 600 trials (~510 standard, ~90 deviant) were collected for each block. All stimuli were normalized for RMS amplitude and presented from a single speaker in the sound field at a level of 70 dBA measured at the location of the infant’s head. All speech stimuli were edited to durations of 500 ms by replicating or cutting vowel cycles without disrupting the natural onset and offset of the sounds.

**Fig. 1:**
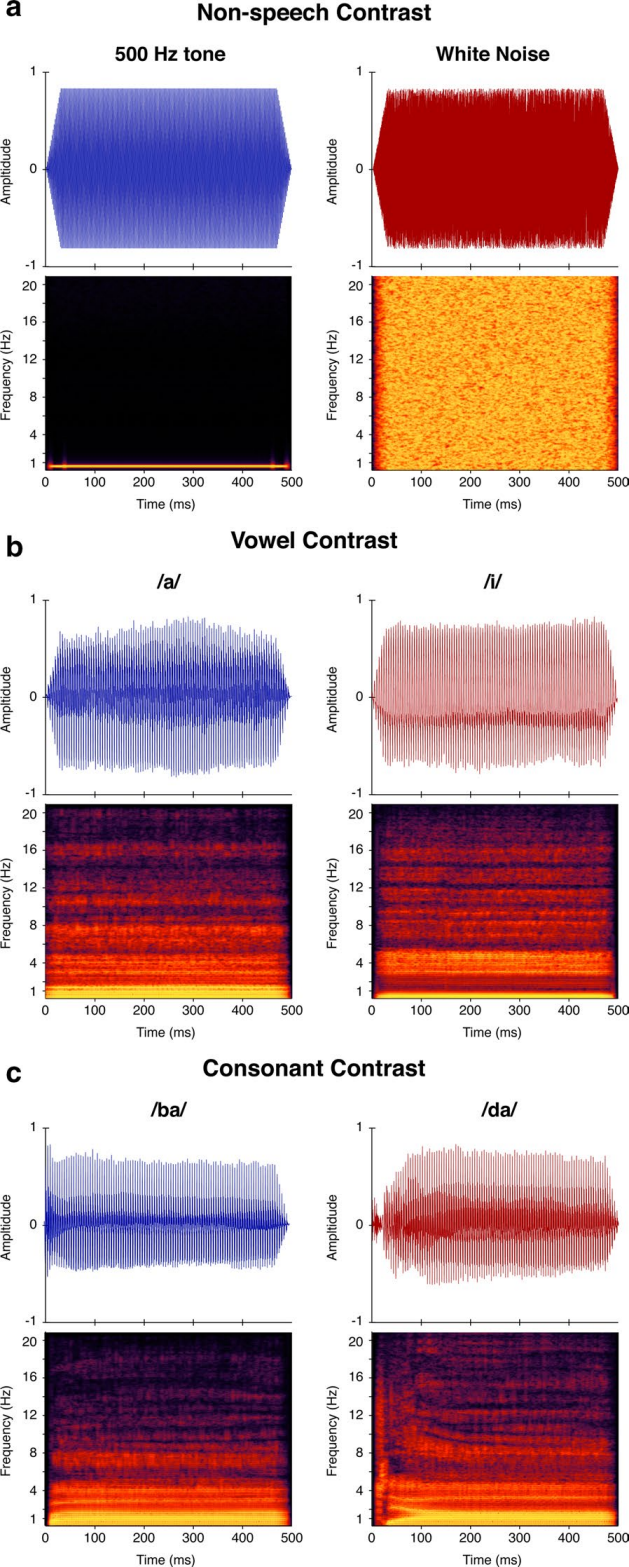
Time-amplitude waveforms (*upper plots*) and time–frequency spectrograms (*lower plots*) for each stimulus. Stimuli are grouped by contrast condition: **a** non-speech (noise-tone), **b** vowel (/i/–/a/), and **c** consonant (/da/–/ba/). The time–amplitude waveforms are plotted in *blue* for the standard stimuli (tone, /a/, and /ba/) and in red for the deviant stimuli (noise, /i/, and /da/)

We chose three contrasts that represent increasing levels of difficulty and developmental emergence for auditory discrimination in young children; these include a non-speech contrast, a vowel contrast, and a consonant contrast (Fig. 1). The order of contrast presentation was randomly selected prior to each recording session. The non-speech contrast is considered the least difficult to discriminate in young children, and consisted of a 500 Hz pure tone (standard) and a white-noise burst (deviant) each 500 ms in duration with 30 ms linear ramping at both onset and offset. The vowel contrast consisted of two naturally produced vowel sounds /a/ (“ah”) and /i/ (“ee”) as the standard and deviant stimuli, respectively. This vowel contrast is considered more difficult to discriminate than the non-speech contrast, and is one of the earliest to emerge in behavioral discrimination tasks [28–30]. The consonant contrast consisted of two naturally spoken consonant–vowel (CV) sounds /ba/ (“bah”) and /da/ (“dah”) as the standard and deviant stimuli, respectively. This contrast is considered the most difficult of the three and tends to emerge later in behavioral discrimination tasks than the vowel contrast. The non-speech and vowel contrasts were completed for all 24 infants, but the consonant contrast was only completed for 17 of the 24 infants.

### EEG procedure

Infants were placed in a comfortable bassinet or in a parent’s lap in a quiet, dim room to induce or aid sleeping during the test session. The rocker’s motion was not active during the EEG recordings, but was active during EEG preparation or during breaks if the infant appeared to be waking. Eleven Ag/AgCl electrodes were placed on the scalp according to the international 10–20 system (F5, Fz, F6, C5, Cz, C6, P5, Pz, P6, M1, and M2) and were referenced to the nasion. An additional bi-polar recording channel was placed on the lateral canthus of the right eye and referenced to the superior orbit to monitor eye movement and waking. Continuous EEG was recorded with a sampling rate of 1000 Hz and filtered from DC-100 Hz during each experimental block using a Synamps2 EEG amplifier (Compumedics-Neuroscan, Charlotte, NC).

### EEG signal processing

All signal processing was conducted in Matlab R2015b (Mathworks, Natick, MA) using the Statistics toolbox with custom tools and the EEGLAB toolbox [31]. Prior to analysis of the experimental trials, the EEG data were filtered from 2 to 50 Hz (zero-phase, FIR, 24 dB/octave). This frequency range includes frequencies associated with early sensory and perceptual evoked potentials and with a high-pass filter above the range of the slow-wave activity during sleep. During the EEG recordings, sufficient EEG was captured before and after each block so that evoked responses were not distorted by the filter edges. Each channel (excluding the eye channel) was then re-referenced to the common average of all 11 data channels. Each contrast block was segmented into epochs from −500 to 1500 ms around each stimulus onset, and baseline corrected to the pre-stimulus interval. Trials with activity greater than 2.5 standard deviations from the mean of the joint-probability distribution of trial amplitudes at each channel were considered as containing artifacts and were rejected from further analysis.

### Channel selection

**Fig. 2:**
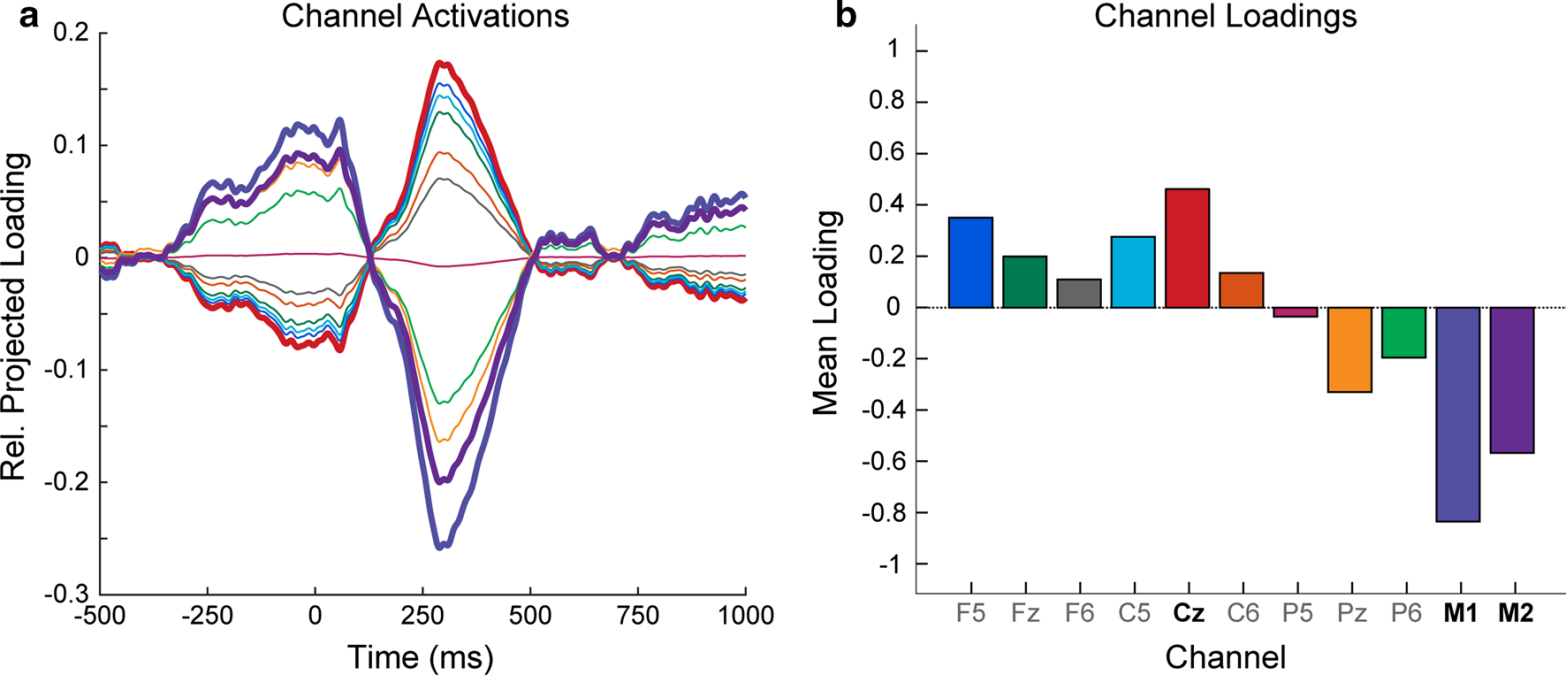
Mean spatial PCA results; **a** channel activations were computed by projecting the selected eigenvectors onto the original channel space by multiplying the eigenvectors with the original input data (bootstrapped differences), and **b** channel loadings were computed as the sum of the mean of the squared, retained eigenvectors for each of 11 scalp electrodes. Electrodes Cz, M1, and M2, which have the largest loadings in opposite polarity, are shown with *thicker lines*; *line color* of the activation in **a** corresponds with *bar color* in **b**

The first goal of this experiment was to determine an optimal recording montage for routine clinical application. We performed a spatial principal components analysis (PCA) on the bootstrapped difference estimate (n = 1001 bootstraps performed individually for each channel) between all standard and deviant trials. Difference estimates for each subject were normalized and concatenated for a group-level PCA. Components were sorted in descending order by the percentage of variance accounted for (pvaf) in the total data. The top components accounting for a cumulative variance of at least 90% were projected onto the channel space (Fig. 2a), and relative magnitudes were then computed as the sum of all trials. This results in a set of “loadings” for each EEG channel (Fig. 2b). Results of this analysis revealed three electrodes with consistently high loadings in all 24 participants: Cz, M1, and M2. Further, the loadings for each of the mastoid channels were negative while the more superior channels had positive loadings. This reflects the polarity inversion of the auditory dipole being above the axial plane of the mastoid electrodes and below the axial plane of the superior electrodes, and confirms the standard practice of recording auditory ERPs from Cz referenced to the mastoids. Taken together, we determined that Cz, referenced to linked mastoids (M1 + M2), would optimize differences in the experimental paradigm. Therefore, the continuous data were re-referenced to the linked mastoids, and only the Cz channel was retained for further analysis.

### Feature extraction

The second and primary goal of this experiment was to examine the spectral-temporal features of the mismatch response (MMR) in infants during sleep. We hypothesized that exogenous auditory processing during sleep would occur in separable EEG frequency bands, and that spectral-temporal features in these bands would reveal responses that indicate the detection of a deviant stimulus [15]. Although we ultimately seek to define such features for individuals, we focus this analysis on group-level effects to better understand the general processes underlying the MMR. To this end, we implemented a modified multi-dimensional scaling (MDS) of the time–frequency transformed EEG trials [32]. This analysis borrows from the DISTATIS method for the analysis of multiple distance matrices [33, 34]; where, here, distances refer to estimated differences in the deviant and standard responses in the time–frequency domain.

### Pre-processing and time–frequency transformation

Each experimental block (i.e., contrast) from each subject was processed separately, and was defined as a 2-dimensional matrix of EEG voltages with size [I × J], where I is the number of trials and J is the number of time points in each trial. Each block was centered and whitened along the Ith dimension via PCA, retaining all eigenvectors explaining at least 0.01% of the total variance. The continuous wavelet transform (CWT) was applied separately to each whitened trial using a 6-cycle Morlet wavelet with 128 log-spaced scales and with center frequencies from 1.94 to 48.40 Hz. The CWT results in a complex, 3-dimensional tensor of size [I × J × K], where K is the number of scales (K = 128) in the transform, and each point includes both a real and imaginary component. After transformation, the time window for each trial was truncated to a range of −100 to 700 ms around the stimulus onset, and the pre-stimulus mean (−100 to 0 ms) was then subtracted from each trial. The time window truncation ensures that all time–frequency results are outside of the cone-of-influence; that is, that the data are not susceptible to edge artifacts. Trials with total squared magnitudes greater than 2.5 standard deviations from the mean were then rejected from the block.

### Computing the MMR_TF_

The probabilistic time–frequency mismatch response (*MMR*_*TF*_) was defined as a probability of the complex magnitude of the estimated mean of the deviant (D) minus standard (S) trials being greater than zero:

1 $$\varvec{MMR}_{{\varvec{TF}}} \mathop = \limits^{\text{def}} P\left( {\left( {\overline{D - S} } \right) > 0} \right)$$

The estimated mean (*D minus S bar*) was computed by a bootstrap difference procedure (*n*-*boots* = 1001) that estimates both the mean difference and the probability of a difference being greater than 0 (i.e., deviant ≠ standard). This method borrows from and extends the methods described for the integrated mismatch negativity (MMN_i_) by Ponton et al. [35]. The estimated mean, ***M***, is computed by randomly selecting one deviant trial and one standard trial (with replacement) during each bootstrap iteration and taking the difference, and is computed as:

2 $${\varvec{M}} = \left\|\frac{1}{n}\mathop \sum \limits_{1}^{n} d_{rd} - s_{rs}\right\|^{2}$$

where *n* is the number of bootstraps (1001), *d*_*rd*_ is a randomly selected deviant trial, and *s*_*rs*_ is a randomly selected standard trial. The term, $\left\| {} \right\| ^{2}$, indicates the squared complex modulus of the mean difference response. The estimated mean error, ***E***, is computed by subtraction of any two randomly selected trials without regard to type (i.e., ignoring standard or deviant):

3 $${ \varvec{E}} = \left\|\frac{1}{n}\mathop \sum \limits_{1}^{n} t_{ra} - t_{rb}\right\|^{2}$$

where *t*_*ra*_ is any random trial from the entire block and *t*_*rb*_ is also any random trial from the entire block. The two results, ***M*** and ***E***, are each real matrices of size [K × J], where J is the number of time points and K is the number of scales. To estimate the probability of a difference, ***M*** and ***E*** were vector normalized across scales, such that the squared sum of all points was equal to one for each scale:

4 $$\varvec{MM}_{\varvec{k}} = \frac{{M_{k} }}{{\sqrt {\mathop \sum \nolimits_{j = 1}^{J} M_{j,k}^{2} + \mathop \sum \nolimits_{j = 1}^{J} E_{j,k}^{2} } }}$$

5 $$\varvec{EE}_{\varvec{k}} = \frac{{E_{k} }}{{\sqrt {\mathop \sum \nolimits_{j = 1}^{J} M_{j,k}^{2} + \mathop \sum \nolimits_{j = 1}^{J} E_{j,k}^{2} } }}$$

where *k* is the Kth scale, *j* is the the Jth point, and ***MM*** and ***EE*** are the normalized means estimates. Therefore, the denominator term reads as the square root of the sum of all squared points in both ***MM*** and ***EE*** for scale *k*. This scale normalization has the effect that all difference points in the time–frequency plane are equated for the energy differences at each scale (e.g., low frequencies naturally have more power over time than higher frequencies). The joint cumulative density function (CDF) for all points in both ***MM*** and ***EE*** was then computed via kernel density estimation with automatic bandwidth selection [36] which we denote as:

6 $$C = CDF(\varvec{MM}{\cup }\varvec{EE})$$

Finally, the probability function for $\overline{D - S}$ is defined by replacing values in ***MM*** with the probability of that value from *C*:

7 $$P\left( {\left( {\overline{D - S} } \right) > 0} \right)\mathop = \limits^{\text{def}} \varvec{MM} \to C$$

Therefore, Eq. 1, which defines the *MMR*_*TF*_, is in turn defined by the joint probability of the true estimation (***MM***) and the error estimation (***EE***), and has a size of [K × J].

### Group-level analysis

The aim of the group-level analysis was to extract and identify the spectral-temporal features that best explain differences between deviant and standard trials, and that may differentiate variances attributed to contrast difficulty. To achieve this aim, the *MMR*_*TF*_ results from each subject and contrast were treated as independent “studies” in a modified multi-dimensional scaling (MDS) analysis [32, 33].

### Spectral and temporal cross-products matrices

Each *MMR*_*TF*_ was treated as a separate distance table and transformed into two cross-products matrices (as in MDS) for the spectral and temporal dimensions, respectively:

8 $$CP_{F} = \varvec{MMR}_{{\varvec{TF}}} *\varvec{MMR}_{{\varvec{TF}}}^{{\text{T}}}$$

9 $$CP_{T} = \varvec{MMR}_{{\varvec{TF}}}^{{\text{T}}} *\varvec{MMR}_{{\varvec{TF}}}$$

where *CP*_*F*_ is the cross-product matrix for the spectral (frequency) representation and *CP*_*T*_ represents the temporal (time) representation. The superscript, T, denotes the transpose.^3^ The centering matrix for each CP was defined as

10 $$\Xi = {\mathbf{I}} - \varvec{l} {\mathbf{m}}^{\text{T}}$$

where **I** is the identity matrix for *CP* with a size of [*n* × *n*], and where n is equal to the size of the dimension in *CP* (i.e., *n* is the number of scales represented in *CP*_*F*_ or the number of points represented in *CP*_*T*_). The term ***l*** **m** ^T^ represents the “mass” contribution for each point in *CP*, where ***l*** is a vector of ones with a size of [*n* × 1] and **m** is mass vector of size [*n* × 1] and whose sum is equal to 1. We set each element of the mass vector to be equal, so each value of **m** was equal to 1/*n*. The superscript, T, denotes the transpose of **m**. The centering matrix was then applied to each CP as:

11 $$\widetilde{CP}_{F} = - \frac{1}{2}\Xi _{F} CP_{F}\Xi _{F}^{\text{T}}$$

12 $$\widetilde{CP}_{T} = - \frac{1}{2}\Xi _{T} CP_{T}\Xi _{T}^{\text{T}}$$

where the subscripts *F* and *T* denote the spectral and temporal domains, respectively, and the superscript T denotes the transpose. Finally, each CP matrix is normalized by its first eigenvalue (*λ* ^1^) and denoted by the symbol **S**:

13 $${\mathbf{S}}_{\varvec{F}} = \lambda_{F}^{1 - 1} \times \widetilde{CP}_{F}$$

14 $${\mathbf{S}}_{\varvec{T}} = \lambda_{T}^{1 - 1} \times \widetilde{CP}_{TT}$$

### Joint DISTATIS

The goal of the modified DISTATIS analysis was to identify a set of weights, denoted **W**, which define the best latent representation of the *MMR*_*TF*_. In our case, we have two separated dimensions (spectral and temporal) represented as normalized cross-products matrices (**S**_*F*_ and **S**_*T*_) for each *MMR*_*TF*_. We therefore define two sets of weights, **W**_*F*_ and **W**_*T*_, for each dimension. Each set of weights was computed via eigendecomposition of a compromise matrix, **C**, for each dimension. The compromise matrix was computed as a weighted mean of all **S** matrices of the dimension. The mean was weighted in that separate means were first computed for each contrast condition (non-speech, vowel, and consonant), and the final compromise was computed as the mean of the individual condition compromises. Therefore, the condition compromise matrices were defined as:

15 $$C_{F}^{d} = \frac{1}{N}\mathop \sum \limits_{n = 1}^{N} {\mathbf{S}}_{{F_{\varvec{n}} }}^{d}$$

16 $$C_{T}^{d} = \frac{1}{N}\mathop \sum \limits_{n = 1}^{N} {\mathbf{S}}_{{T_{\varvec{n}} }}^{d}$$

where the superscript *d* refers to members of the contrast condition, and N is the number of members in that condition. In our case, N was equal to 24 for the non-speech and vowel contrasts, and 17 for the consonant contrasts. The final compromise for each dimension was computed as:

17 $${\mathbf{C}}_{F} = \frac{1}{\varvec{D}}\mathop \sum \limits_{{\varvec{d} = 1}}^{\varvec{D}} C_{F}^{d}$$

18 $${\mathbf{C}}_{T} = \frac{1}{\varvec{D}}\mathop \sum \limits_{{\varvec{d} = 1}}^{\varvec{D}} C_{T}^{d}$$

where D is the number of contrasts (D = 3) and *d* refers to the condition compromise matrices. That is, each final compromise is simply the mean of the condition compromises. Eigendecomposition of the final compromise matrices result in a set of eigenvectors, ***w***, and a corresponding set of eigenvalues, ***λ*** for each compromise. The eigenvalues can be used to determine the percentage of variance accounted for (*pvaf*) by each eigenvector (as in singular value decomposition), where

19 $$pvaf = \frac{\sqrt \lambda }{{\sum {\sqrt \lambda } }}$$

The final weights for each dimension were selected by applying a threshold of 0.01, such that only those eigenvectors explaining at least 1% of the total variance were retained. The retained eigenvectors were normalized such that the sum of the squared values was equal to one for each vector:

20 $${\mathbf{W}}_{F}^{\varvec{U}} = \frac{{w_{F}^{u} }}{{\mathop \sum \nolimits_{u = 1}^{U} \sqrt {w_{F}^{u2} } }}$$

21 $${\mathbf{W}}_{T}^{\varvec{V}} = \frac{{w_{T}^{v} }}{{\mathop \sum \nolimits_{v = 1}^{V} \sqrt {w_{T}^{v2} } }}$$

where *U* and *V* refer to the number of retained eigenvectors in *F* and *T*, respectively; and **W** denotes the final set of weights for the subscripted dimension. The first eigenvalue, *λ* ^1^, for each dimension was used to compute the relative contribution (*rc*) of each dimension to explaining the total variance of the *MMR*_*TF*_:

22 $$rc_{F} = \frac{{\lambda_{F}^{1} }}{{\lambda_{F}^{1} + \lambda_{T}^{1} }}$$

23 $$rc_{T} = \frac{{\lambda_{T}^{1} }}{{\lambda_{F}^{1} + \lambda_{T}^{1} }}$$

### Data projection

Having identified a set of weights for each dimension we assessed the contribution of each *MMR*_*TF*_ to a *joint compromise projection* (${\mathbb{P}}$) of the spectral and temporal dimensions, and each *MMR*_*TF*_ member to its respective condition compromise. In this way, we represent ${\mathbb{P}}$ as a weighted sum of the *MMR*_*TF*_ dimension after a whitening transform by the weights, **W**, as follows:

24 $${\mathbbm{p}}_{F} \left( {\varvec{MMR}_{{\varvec{TF}}} } \right) = {\mathbf{W}}_{F} \times {\mathbf{W}}_{F}^{{\mathbf{T}}} \times \varvec{MMR}_{{\varvec{TF}}}$$

25 $${\mathbbm{p}}_{T} \left( {\varvec{MMR}_{{\varvec{TF}}} } \right) = {\mathbf{W}}_{T} \times {\mathbf{W}}_{T}^{{\mathbf{T}}} \times \varvec{MMR}_{{\varvec{TF}}}^{{\mathbf{T}}}$$

where ${\mathbbm{p}}\left( {MMR_{TF} } \right)$ refers to the projection of the *MMR*_*TF*_ into the dimension’s compromise space (via whitening). It is important to note that each individual *MMR*_*TF*_ was whitened by a set of weights computed from the group-level analysis. The joint projected compromise was then computed as:

26 $${\mathbb{P}} = \left( {{\mathbbm{p}}_{F} \left( {\varvec{MMR}_{{\varvec{TF}}} } \right)*rc_{F} } \right) + \left( { {\mathbbm{p}}_{T} \left( {\varvec{MMR}_{{\varvec{TF}}} } \right)*rc_{T} } \right)^{\text{T}}$$

where * denotes dot multiplication of each projection point with the dimension’s relative contribution, *rc*, from Eqs. 22 and 23. The superscript T refers to the transpose.

After projecting each *MMR*_*TF*_, the condition-level compromise was computed as the mean of all members in the contrast condition:

27 $${\mathbbm{g}}^{d} = \frac{1}{M}\mathop \sum \limits_{m = 1}^{ M} {\mathbb{P}}_{m}^{d}$$

where *M* is the number of *MMR*_*TF*_ responses in the contrast condition, *d* (M = 24 for non-speech and vowel contrasts, and M = 17 for the consonant contrast). Finally, the group-level joint compromise, ${\mathbb{G}}$, was computed as the mean of the condition-level compromise projections:

28 $${\mathbb{G}} = \frac{1}{D}\mathop \sum \limits_{d = 1}^{ D} {\mathbbm{g}}_{d}$$

where *D* is the number of conditions (*D* = 3). The values in both G and g are normalized values between 0 and 1, which represent a *relative probability* (RP) of a difference response for each time–frequency point in the plane (i.e., relative to each other point in the plane).

### Quasi-likelihood estimation

In order to quantify the likelihood that an RP value is significant *on average,* we derived a pseudo M-estimator from the joint compromise matrices. M-estimators define a broad class of statistical estimators that minimize functions of the data (e.g., least-squares estimation, and maximum likelihood estimation). In this case, each *MMR*_*TF*_ is defined as a probability function from Eq. 1, and the ${\mathbbm{p}}\left( {MMR_{TF} } \right)$ projections were computed by a PCA-based whitening function resulting in a normalized relative probability. Because PCA inherently minimizes these functions, we interpret the joint compromise as the “best representation” of this minimization. However, because we are combining more than one “best representation” (i.e., the weighted sum of two minimized functions over two separate dimensions), and each of these representations can contain more than two vectors (i.e., we allow each minimization function to exist in a multi-dimensional space), the representations do not satisfy all properties of a robust M-estimator. Further, combining the dimensions requires projecting the minimized data into an inflated, three-dimensional space with unit-less values, which can make interpretation difficult.

To resolve this issue of interpretation, we mapped the inflated projection, ${\mathbb{G}}$, onto a probability function of ${\mathbb{G}}$, derived from the three condition compromise projections. We first define a minimum bound in the time–frequency plane as the maximum value for each time–frequency point from each of the three compromise matrices:

29 $$\varvec{B}_{ij} \mathop = \limits^{\text{def}} \mathop {\hbox{max} }\limits_{ij \in D} {\mathbbm{g}}_{ij}^{d}$$

where ***B*** represents the minimum bound, and the max term indicates that each time–frequency point *ij* in ***B*** is defined by the maximum value from each of the three condition compromise matrices, where the number of conditions is denoted by *D* and each condition in the set is denoted by *d*. The likelihood is then defined as

30 $$qLE\mathop = \limits^{\text{def}} {\mathbb{G}} \to CDF\left( \varvec{B} \right)^{2}$$

where *qLE* refers to a quasi-likelihood estimate, and takes on a value between 0 and 1, and $CDF\left( \varvec{B} \right)^{2}$ is the squared cumulative density function of the minimum bound, ***B***, as computed by kernel density estimation with automatic bandwidth selection [36]. We interpret the *qLE* as the likelihood that an observation in any joint projected RP value represents a true difference between the deviant and standard responses relative to other observations in the time–frequency plane. The *qLE* is a single [K × J] matrix, which we essentially treat as a likelihood map of the relative probabilities *for these experimental conditions*; that is, it tells us where in the time–frequency plane we are most likely to observe a difference response for any of the three tested contrasts.

Given that the *qLE* represents a probability function, we sought to identify a threshold that can be used for feature extraction and group-level comparisons. Based on pilot analyses of these data, we found that a *qLE* threshold of 0.8 provided enough headroom to compare differences between extracted features, and was low enough to reveal consistently similar features between all participants. We suspect that this threshold may vary for different experimental procedures or populations. For our purposes, we applied a *qLE* threshold of 0.8 to each *MMR*_*TF*_ result. It is certainly possible that some features of interest may be overlooked with this threshold, however, results of this experiment provide some confirmation that this approach reveals consistent features. Future studies using a variety of experimental manipulations will be needed to fully resolve the optimal thresholding procedure.

## Results

### Group-level joint compromise, ${\mathbb{G}}$

**Fig. 3:**
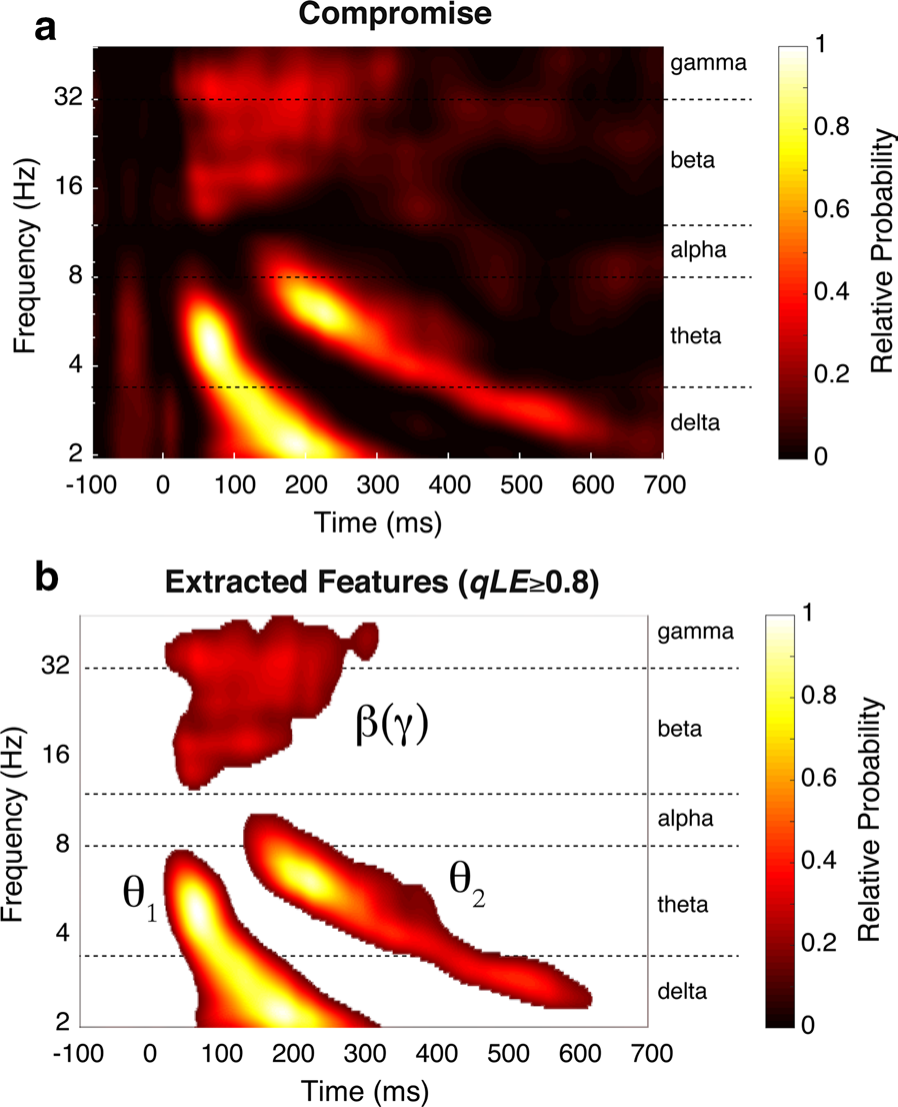
The projected *MMR*_*TF*_ compromise (**a**) and extracted compromise features (**b**). The projected *MMR*_*TF*_ compromise is shown with time in milliseconds along the abscissa, and frequency in Hz along the ordinate. The relative probability (RP) for the compromise is displayed as a heat map defined by the *colorbar*, which has been thresholded at *qLE* ≥ 0.8 in **b** to reveal the three features: beta–gamma, theta-1, and theta-2

The group-level joint compromise is shown in Fig. 3. To identify the spectral-temporal features, we set a threshold on the *qLE* at 0.8, which reveals three general features of interest in the time–frequency plane. The first prominent feature is a burst of activity that begins in the theta band and sweeps downward into the delta band over time. This sweeping burst has two centroids, or peaks, at the beginning and end of the downward sweep. The first centroid appears at 4.7 Hz with a peak latency of 66 ms after stimulus onset, and the second centroid appears at 2.2 Hz with a peak latency of 183 ms. The second feature appears as another downward sweep with slightly higher frequencies and later latencies than the first feature. As before, two centroids define the beginning and end of this sweep with the first centroid at 6.2 Hz with a peak latency of 215 ms and the second centroid at 2.7 Hz with a peak latency of 526 ms. These two features are denoted as theta-1 and theta-2. These theta responses correspond to the temporal phase modulated (PM) theta component (theta-1) and the frontal amplitude modulated (AM) theta component (theta-2) [24, 37, 38].

The third feature in the compromise appears as a large cloudy region spanning the beta (12–30 Hz) and gamma (30–50 Hz) bands. The temporal distribution of this gamma-beta cloud also appears to align with the temporal distribution of the theta-1 response, described above. This alignment is confirmed by comparing the temporal distributions of the gamma-beta and theta-1 responses for each condition (see Fig. 5 and “[Sec Sec18]” section, below). Such alignment corroborates evidence for a cross-frequency coupling (CFC) effect for information binding [39, 40].

### Condition-level joint compromises, $\mathbbm{g}^{\text{d}}$

**Fig. 4:**
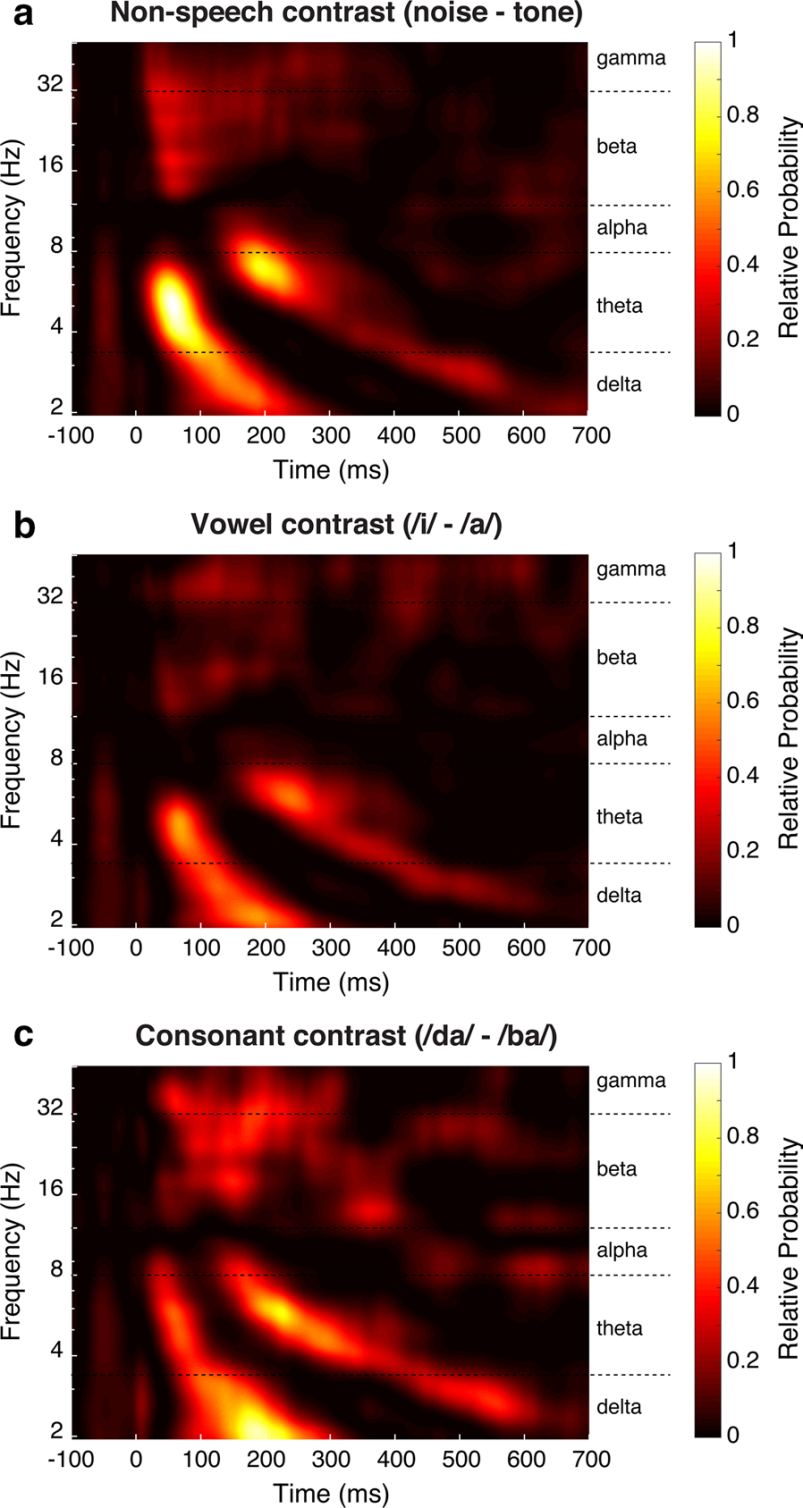
*MMR*_*TF*_ compromise projections by condition: **a** non-speech (noise-tone), **b** vowel (/i/–/a/), and **c** consonant (/da/–/ba/). Each projection is shown with time in milliseconds along the abscissa, and frequency in Hz along the ordinate. The relative probability for the compromise is displayed as a heat map defined by the *colorbar*

The condition-level joint projections are shown in Fig. 4. The three features (theta-1, theta-2, and beta–gamma) of the group-level compromise are apparent in each of the condition-level projections, but with some notable differences upon visual inspection. For example, the theta-1 power appears to peak with a higher frequency and earlier latency for the non-speech and vowel contrasts compared to the consonant contrast.

The beta–gamma feature in the vowel contrast appears to be sparser and with less power than the other contrasts. There also appears to be an overall latency shift between the three features such that the non-speech contrast is earliest, followed by the vowel contrast, and then the consonant contrast. We sought to quantify these feature differences by comparing the centroids of each feature across contrast conditions.

We defined condition-feature centroids as the joint maximum of the temporal and spectral distributions for each feature. To derive these distributions, we created separate two-dimensional masks for each of the three features defined by *qLE* ≥ 0.8. First, a mask H was created from the *qLE* values as:

31 $${\text{H}} = \left\{ {\begin{array}{*{20}c} {0,} & {qLE < 0.8} \\ {1,} & {qLE \ge 0.8} \\ \end{array} } \right.$$

**Fig. 5:**
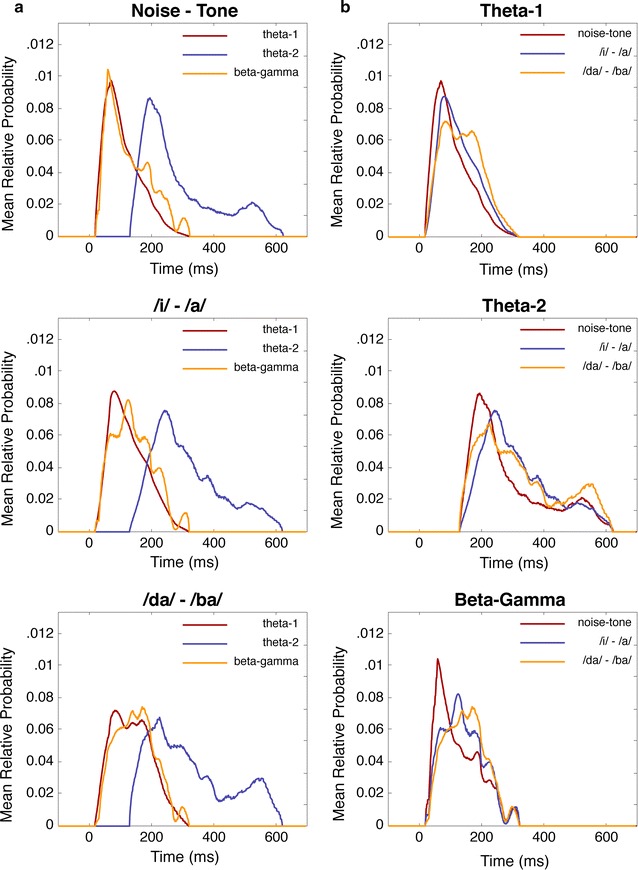
Mean temporal probability distributions for each extracted feature (beta–gamma, theta-1, and theta-2). In **a** the distributions are plotted by condition to highlight the differences in each feature; whereas in **b** the distributions are plotted by feature. In each plot, time in milliseconds is represented along the abscissa and the mean probability is plotted along the ordinate

**Fig. 6:**
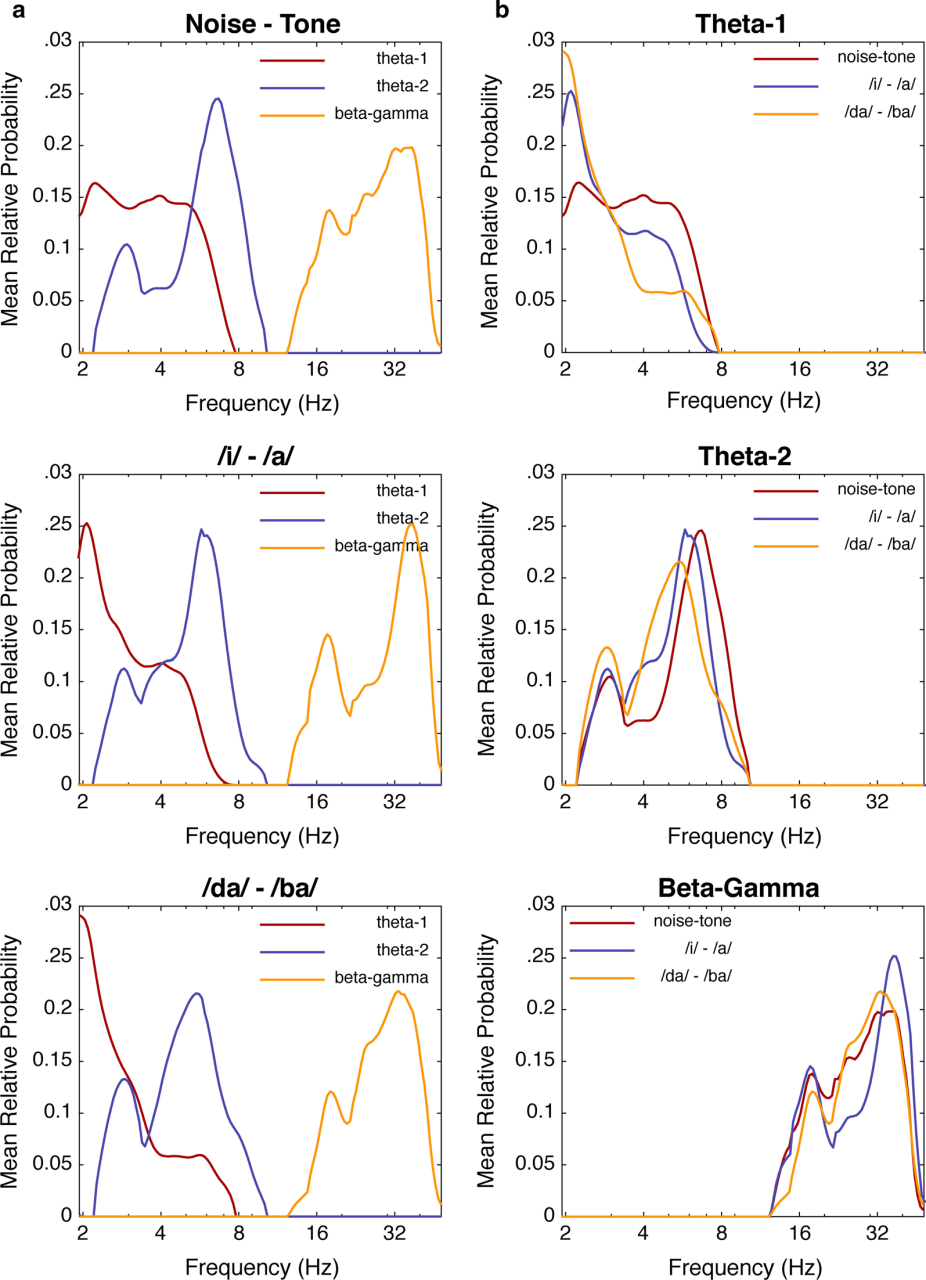
Mean spectral probability distributions for each extracted feature (beta–gamma, theta-1, and theta-2). In **a** the distributions are plotted by condition to highlight the differences in each feature; whereas in **b** the distributions are plotted by feature. In each plot, frequency in Hz is represented along the abscissa and the mean probability is plotted along the ordinate

Separate masks were then created for each feature (*d*) by setting only those values bound within the feature to 1. Each feature mask (${\text{h}}^{d}$) was then multiplied with each condition-level projection ($\mathbbm{g}^{d}$), and the temporal (subscripted T) and spectral (subscripted F) distributions of the feature were then computed as the mean across the respective dimension:

32 $$y_{T}^{d} = \frac{1}{J}\mathop \sum \limits_{j = 1}^{J} \left( {{\text{h}}_{j}^{d} *\mathbbm{g}_{j}^{d} } \right)$$

33 $$y_{F}^{d} = \frac{1}{K}\mathop \sum \limits_{k = 1}^{K} \left( {{\text{h}}_{k}^{d} *\mathbbm{g}_{k}^{d} } \right)$$

where *y* denotes the mean RP distribution for the subscripted dimension and superscripted feature. Condition-feature distributions are shown in Figs. 5 and 6. Interestingly, the temporal distributions for the theta-1 and beta–gamma features are nearly identical in each of the conditions, which further supports a CFC effect between the theta-1 and gamma–beta responses.

**Fig. 7:**
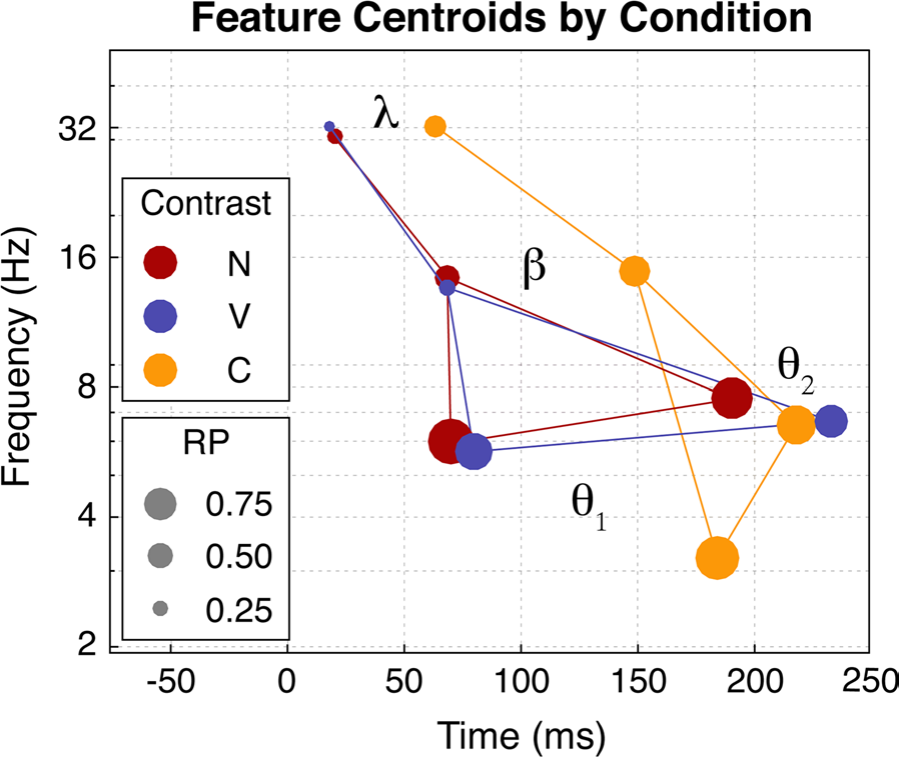
Feature centroids by condition. The spectral-temporal center of each feature is plotted as a *circle*, separately for each condition. Conditions are displayed in three different colors: *red* (labeled “N”) is the non-speech (noise-tone) condition, *blue* (labeled “V”) is the vowel (/i/–/a/) condition, and *orange* (labeled “C”) is the consonant (/da/–/ba/) condition. The *dotted lines* simply connect each condition’s centroids for visualization purposes. The relative probability at each centroid is denoted by change in size of the circle, as indicated in the *lower* key

Feature centroids for each condition were selected by conducting a search of the local maxima within each feature. This search revealed that the gamma–beta feature consists of two distinct maxima, a gamma and beta centroid, and that the theta-2 feature consisted of two distinct maxima corresponding to the approximate onset (theta-2) and offset (theta-2b) of the feature. The temporal and spectral distributions in Figs. 5 and 6 confirm that the gamma and beta centroids are distinct components, and that the theta-2b centroid appears to be a distinct component. The latency, frequency, and RP values for each centroid are listed in Table 1, and represented graphically in Fig. 7. We denote the second theta-2 centroid as theta-2b in Table 1, but it is not depicted in Fig. 7.

**Table 1:** Centroid frequency, latency, and relative probability for each spectral-temporal feature (gamma, beta, theta-1, theta-2, and theta-2b) and each contrast condition: non-speech (noise-tone), vowel (/i/–/a/), and consonant (/da/–ba/)

	Noise-tone	/i/–/a/	/da/–/ba/
*Gamma*			
Frequency (Hz)	33.94	35.71	35.71
Latency (ms)	22	20	59
Relative probability	0.25	0.09	0.38
*Beta*			
Frequency (Hz)	17.56	16.28	18.47
Latency (ms)	56	56	147
Relative probability	0.37	0.16	0.40
*Theta-1*			
Frequency (Hz)	5.07	4.70	2.09
Latency (ms)	58	69	187
Relative probability	1.00	0.62	0.92
*Theta-2*			
Frequency (Hz)	7.05	5.91	5.76
Latency (ms)	194	242	225
Relative probability	0.78	0.54	0.76
*Theta-2b*			
Frequency (Hz)	2.98	2.83	2.90
Latency (ms)	481	493	516
Relative probability	0.26	0.20	0.38

## Discussion

Our aim was to examine and describe the spectral-temporal features of mismatch responses in the EEG of sleeping infants from a clinically viable electrode montage. The results of this study provide valuable insight into the neurophysiological mechanisms that underlie processes of prediction and discrimination in the developing brain. Below, we discuss the spectral-temporal features of these mechanisms, and describe a model for how these responses may reflect early speech discrimination.

The group-level compromise shown in Fig. 3 depicts, essentially, the experiment-wise probability of observing a spectral-temporal modulation during any given deviant trial. These spectral-temporal bounds are corroborated by previous findings of theta, alpha, beta, and gamma modulations at similar frequency ranges and latencies in auditory oddball responses. The present results suggest that auditory oddball discrimination, as represented by the *MMR*_*TF*_, occurs as a dynamical process between multiple neural generators spanning the delta, theta, beta, and gamma frequency bands. We suspect the lack of an alpha response is due to sleep state, as alpha is generally associated with shifts in attentional processing while awake.

### Discrimination and contrast difficulty

When comparing the condition-level compromise projections (Fig. 4), we did not anticipate the generally low power in the responses to the vowel contrast (/i/–/a/) when compared to the non-speech (noise-tone) and consonant (/da/–/ba/) contrasts. Based on the ease of behavioral discrimination of /i/–/a/ [28–30] we predicted that the relative probabilities would be quite large and distinct. Rather, responses to the consonant and non-speech contrast were more robust than for the vowel contrast. There are several plausible explanations for this. First, the vowel contrast is composed of a spectral shift (i.e., a shift in vowel formant frequencies), whereas the consonant and non-speech contrasts are composed of both spectral shifts and rapid, aperiodic temporal shifts. It is possible that spectral differences are subtler than the more salient temporal differences, and thus elicit a smaller oddball response. However, this does not explain the ease of behavioral vowel discrimination.

A second explanation for the vowel difference led us to review the work by Saffran et al. [41–43] who have thoroughly examined various aspects of development of language-learning. Their research suggests that language learning occurs as a probabilistic function of sound and sound-pattern exposure. For example, infants learning English respond better to multi-syllabic patterns with stress on the first syllable, as those sounds are more likely to indicate a new word boundary. Functionally, a new word boundary indicates a greater likelihood of new information to be processed, which inherently requires more processing resources. Extended from this, processing resources for vowels, which span much longer time courses than consonants, require fewer resources to identify (relatively) slowly changing features such as formant shifts, which, probabilistically, do not alter the actual information content as extensively as a new word boundary. In this way, the smaller resource allocation for vowel processing would be observed as diffuse spectral power, which was observed here. The longer time course for processing the vowel information accounts for a greater probability of detecting a difference with relatively fewer resources expended, which may explain the ease of behavioral vowel discrimination.

### Spectral-temporal dynamics

**Fig. 8:**
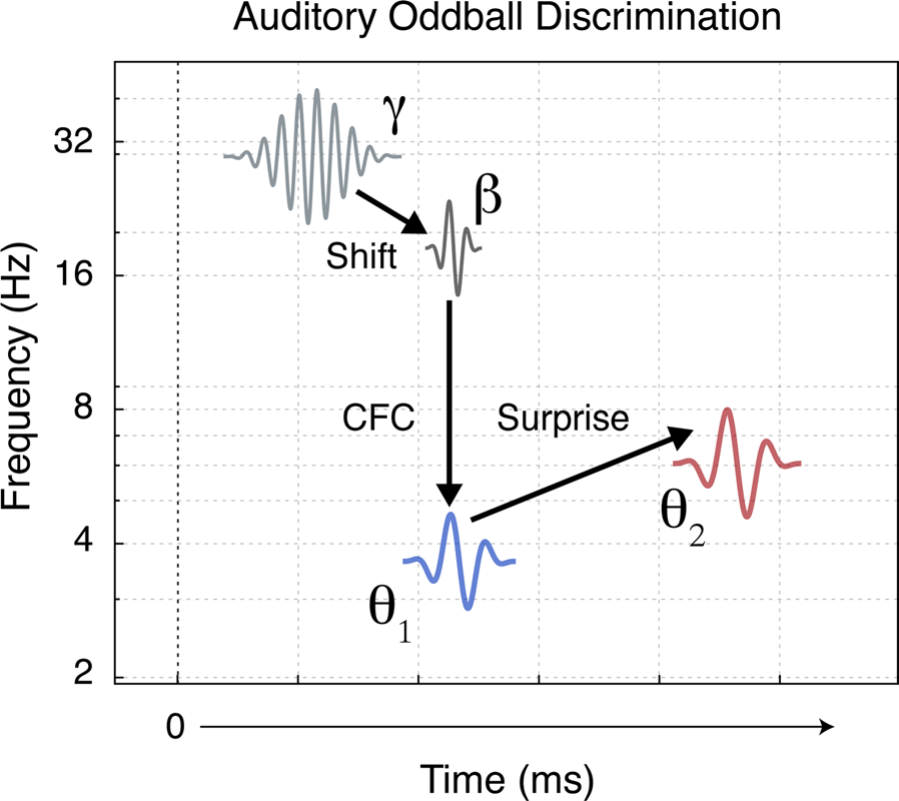
Schematic model of the spectral-temporal dynamics of auditory oddball discrimination. Time from stimulus onset is represented, arbitrarily, along the abscissa. Frequency in Hz is represented along the ordinate. In this schematic, a deviant stimulus initiates a gamma response, which is then shifted into a beta synchronization when the auditory signal differs from some expectation. This gamma-beta wave packet is coupled (CFC) with a theta oscillator, theta-1 (shown in *blue*). An additional theta oscillator, theta-2 (shown in *red*), is activated at a slight delay relative to the beta–gamma burst. The difference between these two theta oscillators reflects the degree to which the actual response differs from the expected response; that is, it reflects some amount of surprise to the deviant sound

The centroids corresponding to each oscillatory component, as shown in Fig. 7, depict a spectral-temporal hierarchy for change detection [44–46], which is represented schematically in Fig. 8. This hierarchy suggests that auditory oddball discrimination is initiated as a gamma burst, which then likely emerges as a spatial amplitude modulation in the beta range (cf., [48]). This gamma–beta wave packet is temporally coupled with an oscillatory theta component, which is followed by a second theta component. The latency of the temporal coupling effect relative to the peak latency of a second theta oscillation reflects some degree of discrimination for the deviant sound. Further, as the latency of the coupling effect increases the coupling frequency of the theta oscillation decreases to frequencies as low as 2 Hz (which was the lower limit of resolution for the spectral dimension). Taken together, this cascading process of modulation converges with evidence that oddball responses are driven by predictive processes that gate incoming sensory information by a measure of surprise (see footnote 3) [47–49].

### Gamma and beta modulation

When applying a conservative threshold of *qLE* ≥ 0.8, the responses in the gamma and beta bands appeared as a consolidated region (Fig. 3b), and are similar to the beta/gamma responses described by Isler et al. [15] and Stefanics et al. [50]. After separating the centroids for the beta and gamma responses (Fig. 7) we observed a distinct pattern of a gamma–beta shift that corroborates findings described in ERP experiments [51, 52] and from local field potentials and single-unit recordings of hippocampal and neocortical pyramidal cells in animal models [39, 53]. Results from animal models suggest that cells initially respond to a stimulus by releasing a burst of activity in the gamma range and then rapidly shift to a burst of slower activity in the beta range (with limitations; see Traub et al. [53]). This gamma–beta shift may define a temporal window for long-range coherence between active components in a neural network, and may determine whether excitatory connections can synchronize at beta frequencies. For example, it is possible that local gamma oscillations act as a gating mechanism by limiting beta synchronization of gamma frequency responses to repeated, or *expected* stimulation. The presentation of a deviant sound would then, presumably, result in a larger gamma frequency response including responses from unexpected regions, and thus from uninhibited cells that are capable of beta synchronization. However, the gamma–beta shift does not, alone, describe the differences observed in an oddball response. For example, there must be something different about this particular shift than a shift that occurs during a standard response.

The speed at which the brain detects a difference in stimuli is quite remarkable, and is a process that begins as early as 20 ms after the stimulus onset. When detecting a deviant stimulus, change responses appear in the gamma band as early as 20 ms for easier contrasts and can be much later, ~60 ms, for more difficult contrasts, representing a wide range of variability for detection latency. The beta centroids appear slightly later at about 50–60 ms for easier contrasts, and can be much later at about 120–150 ms for more difficult contrasts. These beta centroids also correspond with the beta/gamma latencies reported by Isler et al. [15] and Stefanics et al. [50]. One explanation for this very rapid change detection is that these responses reflect neural mechanisms of pattern recognition and predictive processing; processes represented by gamma–beta shifts to changes in expectancy. Evidence for such predictive processing has been eloquently described in a review by Bendixen et al. [49]. In that review, the authors present a comprehensive model of predictive processing that (generally) includes a spectral-temporal hierarchy, such as that described by the results in this and other studies [44, 45, 52, 54]. To better understand this process, we must also account for the changes in lower frequency theta components that correspond to the detection of deviant sound.

### Cross-frequency coupling

Another important feature of the *MMR*_*TF*_ is the concurrent timing of the gamma–beta and theta-1 modulations (see Fig. 5). Such concurrent timing between different frequency components is often described as a cross-frequency coupling (CFC) response (for review, see [39]). CFC is hypothesized as a mechanism for long-range synchrony between distinct, local generators of gamma activity. Schematically, we might think of the lower frequency theta waves as spatial modulators between network components operating in the gamma and beta ranges (e.g., see [47]). With regard to deviance detection, the latency and coherence of CFC may act as a primary gating mechanism for incoming information, where some combination of latency and coupling strength provides a measure of surprise. The coupling latencies for each condition are represented in Fig. 7, which clearly shows a later CFC effect for the more difficult consonant contrast.

To make sense of this process, consider the simplicity of the basic oddball paradigm: one sound is repeated periodically, and a second sound is occasionally played instead; with this being repeated hundreds of times. If beta synchronization occurs via a gamma–beta shift as described above, then we might expect a larger gamma response to a deviant sound, followed by a noticeably larger beta synchronization. The emergence of beta synchronization occurs at some phase relative to a theta oscillator that itself is phase-locked to the expected rate of repetition [52]. It is plausible that the beta wave packet then perturbs this theta oscillator, which we observe as the CFC effect. As the original sound is repeated again, we should observe a habituation effect with each repetition, and we would expect that habituation to be most notable in beta frequency ranges and latencies. In the present study, we did not test for this habituation effect, but previous studies of habituation and refractoriness support this notion [26, 55].

### Theta modulation

We might infer that the observation of a theta-1 modulation is driven by the CFC effect; that is, we observe a theta-1 perturbation concurrent with beta synchronization. However, this inference does not account for the observation of the theta-2 response, nor does the present study allow us to resolve this issue. One explanation is that the theta-2 response occurs as a release from local inhibition of beta synchronization by concurrent gamma oscillations, as described above. Another explanation is that a dedicated theta oscillator responds solely to perturbations of other theta oscillators that are actively processing incoming information. Yet another explanation is that the cause of the theta-2 response is simply not observed by these analysis methods. For example, because the difference estimates were computed in the complex time–frequency plane, we expect to observe spatial AM and PM effects as spectral perturbations, while FM and spectral power effects would be less noticeable. Other experimental methods, such as those that resolve synchronization/de-synchronization effects [51] may be better suited to resolving the catalyst of theta-2.

Fuentemilla, Marco-Pallarés et al. [22, 56] suggested that two distinct theta components, a frontal amplitude modulated component and a temporal phase modulated component, represent operations from distinct neurophysiological mechanisms. Hsiao et al. [23] further suggested that these two theta components might characterize long-range phase synchrony within a temporo-frontal network for change detection. In the present results, theta-1 corresponds with the earlier temporal component and theta-2 with the later frontal component. Comparisons of the condition-level compromises show that the difference between easier and more difficult contrasts is represented by a spectral-temporal shift between these two theta components. We cannot say whether the time–frequency shift between the two theta components is a determinant of phase modulation, or “phase-resetting”, or a consequence thereof; however, we suggest that this time–frequency shift corresponds with such a mechanism.

### Expectancy and surprise

Taken together, a dynamical process of expectancy and surprise^4^ can characterize deviance detection in an oddball paradigm. In this way, the entropy of an oscillating network characterizes the network’s expectation [47, 48]. When the network encounters some amount of surprise relative to the expected information, a perturbation of that network initiates the conditions for dissipating or transferring the unexpected information to higher-order processes. For example, the ability of local gamma responses to initiate beta synchronization may be dependent on some threshold for surprise. Further, this synchronization results in series of phase transitions to lower frequency components, as observed by the CFC effect subsequent to a gamma–beta shift. The actual amount of surprise might then be characterized by the spectral-temporal shift between theta-1 and theta-2. For example, the temporal distributions shown in Fig. 5a support this notion as the difference in the peaks of the theta distributions are closer together for more difficult contrasts, and include a greater likelihood of the response occurring in the lower delta range.

We suggest that the degree of difficulty for detecting a deviant stimulus is represented by a probability function that is constrained by the difference in both frequency and latency of the theta-1 and theta-2 components and by the latency and coupling strength of the beta–gamma and theta-1 components. Specifically, the process of deviance detection in an oddball paradigm can be characterized by a measure that is proportional to the surprise, *S*:

34 $$S \propto - { \log }\left( {\frac{\partial F}{C\partial T}} \right)$$

where $\partial F$ is the probability of a difference in frequency between theta-1 and theta-2, and $C\partial T$ is the probability of a latency difference between theta-1 and theta-2 multiplied by a constant, *C*, which accounts for the latency of the CFC effect. This equation suggests that the spectral-temporal difference between theta-1 and theta-2 reflects the amount of information in the stimulus that was different than expected.

### Experimental and clinical implications

The results reported here have implications for future experimental research utilizing an oddball paradigm, as well as for the clinical application of such methods. A key difference in our results and previous results is that spectral-temporal modulations are defined by a probability function instead of by measures of power or coherence. In this way, the *MMR*_*TF*_ represents a complex wave function that “collapses” upon observation [57]. Considering the *MMR*_*TF*_ as a wave function has implications for how we might interpret results from these experiments. If the wave function represents the probability of an observation, then the actual response during any given trial can appear anywhere within that function. Thus, for any single trial there is a significant amount of uncertainty as to whether such a response *did* occur, but after multiple observations we gather information to support some probability that the response *can* occur.

One interpretation of this probabilistic view is that any single-trial analysis of oddball responses must rely on some a priori information about the underlying system [58]. Fortunately, such an assumption provides a basis of support for implementing machine-learning methods for single-trial analyses. In this study, we chose the modified DISATIS algorithm because, mathematically, this approach acts as a precursor for kernel-based methods such as kernel-PCA, kernel-ICA, and support vector machines. Therefore, defining these responses as a probabilistic wave function provides considerable insight into how a machine learning approach might be implemented in future experiments. Unfortunately, such assumptions also infer a lower limit on the number of observations needed to achieve certainty for the probability function.

A key motivation for this research is the development of objective measures that might be used to assess auditory function in children with pathologies affecting the auditory system [15, 59]. For example, an infant identified with hearing loss must be fit with amplification (e.g., hearing aids) that is tuned to optimize information processing in certain frequency bands; for example, the speech frequencies. Whether an infant can discriminate two speech sounds depends on how well the amplification is tuned, which has direct implications for language-learning [41, 60]. An objective measure such as the *MMR*_*TF*_ might provide a means to assess whether an infant has adequate access to the speech frequencies. From a clinical perspective, we wish to determine whether an infant *can* discriminate between two speech sounds. As another example, children with auditory processing disorders exhibit deficits in beta coupling observed as shifts in beta synchronization frequencies [52, 54]. A measure such as the *MMR*_*TF*_ might provide a means to better classify these deficits, or to provide real-time feedback during assessment. In this case, we wish to determine whether a child *did* discriminate between two sounds. Of course, to better understand these potential applications, further research including these populations will be necessary.

Clinical use of an oddball paradigm such as the MMR/N is often met with skepticism over the validity of results at the individual level [15, 61]. One source of such skepticism is the large variability in the latencies of MMR/N responses analyzed via traditional signal averaging. Indeed the spectral-temporal proximity of the theta-1 and theta-2 responses likely account for much of this variability, as these two components would appear as a mixture in the averaged ERP (cf., [62]). Further, the variability of the response onset—the CFC effect—contributes to the temporal overlap and subsequent smearing in the averaged response. Results reported here support the notion that time–frequency analyses improve the likelihood of detecting and classifying these responses within individuals.

## Conclusions

Even during sleep, an infant’s brain is processing information about the environment and performing computations about that information in an unconscious state. Moreover, these computations reflect subtle differences in acoustic feature processing that are necessary for language-learning. Results from this study suggest that brain responses to deviant sounds in an oddball paradigm follow a cascade of oscillatory modulations. This cascade begins with a gamma response that later emerges as a beta synchronization, which is temporally coupled with a theta modulation, and followed by a second, subsequent theta modulation. The difference in frequency and timing of the theta modulations appears to reflect a measure of surprise; that is, it provides a measure of the error between the information that was expected, and the information that was actually received. These insights into the neurophysiological mechanisms of auditory discrimination provide a basis for exploring the clinically utility of the *MMR*_*TF*_ and other auditory oddball responses.

## Abbreviations

AM: amplitude modulation
CFC: cross-frequency coupling
CWT: continuous wavelet transform
EEG: electroencephalography
ERP: event related potential
FM: frequency modulation
MDS: multidimensional scaling
MMR_TF_: probabilistic time–frequency mismatch response
MMR/N: mismatch response/negativity
PCA: principal components analysis
PM: phase modulation
pvaf: percentage of variance accounted for
qLE: quasi-likelihood estimate

## Footnotes

1 Such tasks need not be limited to only two sounds; however, we limit the discussion here to a traditional oddball paradigm of two contrasting stimuli.

2 While traditionally referred to as the mismatch negativity (MMN), infants exhibit a difference response with positive orientation, which is reflected by the more inclusive use of the mismatch response (MMR) to describe these responses.

3 Note that for all equations a superscripted “T” refers to the transpose of a matrix or vector, whereas the subscripted “*T*” refers to the temporal dimension of the data.

4 In information theory, surprise is defined by the negative logarithm of the expected information content (i.e., the entropy).
